# Seizure treatment in children: can intranasal midazolam be used without an atomizer?

**DOI:** 10.31744/einstein_journal/2025AO1535

**Published:** 2025-08-15

**Authors:** Karini Da Rosa, Cassiano Mateus Forcelini, Débora Miotto Lorenzetti, Matheus Alberto Cella, Marina Camargo Galera, Vítor Alexandre Ferraz de Carvalho e Souza, Rafael Linden, Charise Dallazem Bertol

**Affiliations:** 1 Universidade de Passo Fundo Postgraduate Studies in Human Aging Passo Fundo RS Brazil Postgraduate Studies in Human Aging, Universidade de Passo Fundo, Passo Fundo, RS, Brazil.; 2 Universidade de Passo Fundo Escola de Medicina Passo Fundo RS Brazil Escola de Medicina, Universidade de Passo Fundo, Passo Fundo, RS, Brazil.; 3 Universidade Feevale Pharmacy Course Novo Hamburgo RS Brazil Pharmacy Course, Universidade Feevale, Novo Hamburgo, RS, Brazil.; 4 Universidade de Passo Fundo Pharmacy Course Passo Fundo RS Brazil Pharmacy Course, Universidade de Passo Fundo, Passo Fundo, RS, Brazil.

**Keywords:** Seizures, Status epilepticus, Administration, intranasal, Midazolam

## Abstract

**Objective:**

Convulsive status epilepticus is the most common neurological emergency in children with high morbidity and mortality rates. Intranasal midazolam administration using an atomizer is the preferred treatment method. However, Brazilian pediatric emergencies often require rapid midazolam instillation using syringes. We aimed to compare the plasma midazolam levels in children who received intranasal medication with an atomizer and those who received medication via rapid instillation using only a syringe to treat seizures.

**Methods:**

This exploratory cross-sectional study was conducted between January 2022 and March 2024 at the emergency department of a hospital in Southern Brazil. Thirteen children with tonic-clonic status epilepticus received intranasal midazolam via either an atomizer or syringe, at similar dosages. The peak plasma midazolam concentrations, measured approximately 10 min following intranasal administration, were compared between the groups.

**Results:**

No significant difference was observed in the plasma midazolam concentrations (ng/mL) between the syringe and atomizer groups (median and interquartile range: 148.8 [126.3-293.7] ng/mL *versus* 133.3 [76.4-176.3] ng/mL; p=0.414), as well as in the time from administration to sampling (8.0 [7.75-10.0] min *versus* 9.5 [6.75-10.0] min; p=0.710) or in weight-adjusted dose (0.48 [0.44-0.50] mg/kg *versus* 0.49 [0.47-0.56] mg/kg; p=0.414).

**Conclusion:**

This study is the first to validate the common Brazilian practice of using syringes alone for rapid nasal midazolam delivery during pediatric emergencies. Future studies should elucidate the clinical outcomes of this approach.

## In Brief

Da Rosa et al. compared midazolam plasma levels in children with tonic-clonic status epilepticus intranasally treated with an atomizer or syringe. No significant differences were observed between the groups. This study is the first to validate syringe-only nasal delivery, which is an extremely common practice in Brazilian pediatric emergencies.

## Highlights

■ Intranasal midazolam with an atomizer is a treatment for pediatric status epilepticus.
■ In Brazil, the atomizer is often replaced with a syringe owing to the cost of the device.
■ We compared midazolam plasma levels with and without an atomizer in children with epilepsy.
■ No significant difference was found, supporting the syringe-only use in emergencies.

## Graphical abstract

**Figure:**
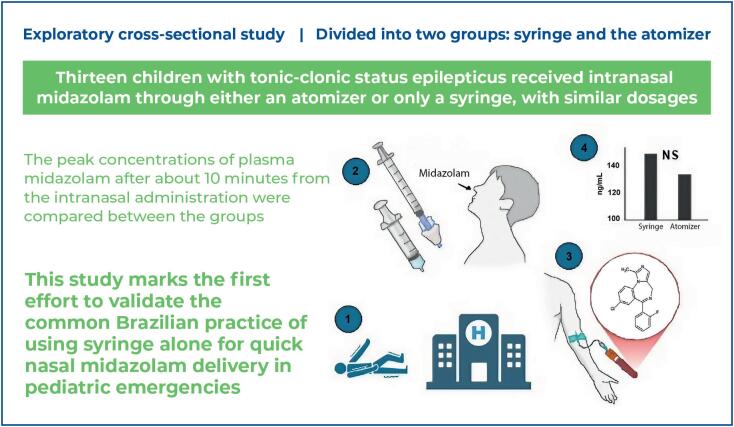

## INTRODUCTION

Convulsive status epilepticus is characterized by tonic-clonic seizures that last for at least 5 min.^(1)^ It is the most frequent neurological emergency in children and carries considerable morbidity and mortality risks. The prognosis worsens with delayed treatment.^(2-4)^

Several studies between 1996 and 2006 highlighted the potential benefits of intranasal midazolam in managing acute seizures in children.^(5-9)^ These pioneering studies involved slowly dripping an intravenous midazolam solution into the nose, typically dividing the dose between nostrils. The rationale is that this approach may increase the effectiveness of midazolam by elevating its availability for seizure treatment, potentially allowing it to cross the blood-brain barrier through the olfactory pathway and systemic circulation.^(10,11)^ However, this increased availability is yet to be conclusively shown.

Mucosal atomization devices (atomizers) have become the preferred method for rapid intranasal midazolam administration for pediatric seizure treatment since their introduction in 2007.^(12)^ The atomizer can enhance absorption by dispersing microdroplets across the nasal mucosa instantly, rather than via slow dripping. However, their high cost limits their use in developing countries. Alternatively, rapid instillation using a syringe has become common practice in Brazilian pediatric emergencies for status epilepticus. A double dose (0.5mg/kg of the intravenous solution at 5mg/mL) is usually prescribed to counterbalance any absorption loss owing to the absence of micro-droplet dispersion.^(12)^ Contrastingly, this practice diverges from earlier studies that suggested the benefits of slow dropping in each nostril.^(5-9)^ In summary, no publications support its efficacy despite this approach being widely used.

In this study, we compared the plasma midazolam levels in children who received intranasal medication with an atomizer and those who received medication via rapid instillation using only a syringe for seizure management.

## OBJECTIVE

To compared plasma midazolam levels in children who received intranasal medication with an atomizer and those who received medication via rapid instillation with only a syringe for treating seizures.

## METHODS

The local Ethics Committee approved this exploratory cross-sectional study in December, 2021 (CAAE: 53719521.5.0000.5342; #6.265.562; *Universidade de Passo Fundo*). Clinical data and plasma samples were collected over two years (from January 2022 to March 2024) in the emergency department of *Hospital São Vicente de Paulo*, Passo Fundo, RS, Brazil. During the first half of 2024, laboratory analyses were conducted at the Toxicology Laboratory of the *Instituto de Ciências da Saúde, Universidade Feevale*, Novo Hamburgo, RS, Brazil.

This study included children aged 0-12 years who were brought to the emergency department by their parents with tonic-clonic status epilepticus. While the children were receiving care, their parents were briefly invited to participate by a member of the research team. After obtaining written consent, the inclusion criteria were promptly evaluated to facilitate patient enrollment. No parents declined participation.

Inclusion criteria were as follows: 1) children aged 0 to 12 years; 2) diagnosis of tonic-clonic status epilepticus; 3) recent known weight reported by parents; 4) ability to obtain a blood sample approximately 10 min (between 6 to 14 minutes) following intranasal midazolam administration; 5) intranasal intravenous midazolam dosage (5mg/mL) at 0.5mg/kg based on reported weight; 6) actual administered midazolam dose ranging from 0.4 to 0.6mg/kg according to weight checks after seizure control; and 7) indication for blood sample collection by the pediatrician for routine investigation of the seizure cause.

No additional venous puncture was performed for this study. We used the opportunity for a clinical indication for blood tests to collect a small amount of blood for laboratory analysis, contingent upon parental consent. Blood samples were immediately processed in a local laboratory to separate 2mL of plasma, which was then stored at -21 °C for further analysis using high-performance liquid chromatography mass spectrometry with an Acquity UPLC BEH C18 column (Waters Corporation, USA).

The ideal timeframe for obtaining blood samples was based on the literature, indicating that the median time to the maximum concentration of intranasal midazolam with an atomizer was 10 min.^(13)^ Furthermore, this study reported considerable concentrations at 5 and 20 min after administration, although it was lower than the peak.^(13)^ Thus, our study protocol defined an acceptable window of 6-14 min following administration for blood sample collection. Similar pharmacokinetic findings were observed in a recent study involving children undergoing sedation for minor surgical procedures.^(14)^

Data were collected according to standard practices without an atomizer during the first half of the study. Eight atomizers (GC Medica, China) were purchased for use in the second half of the study. Finally, the plasma samples and clinical data from both periods were compared.

This exploratory study was the first to evaluate such a comparison. We used the study by Walbergh et al. to define the minimum sample size.^(15)^ They compared the plasma midazolam concentrations obtained by intranasal instillation in the form of drops (without a syringe or atomizer), intravenously administered in children. The difference between the peak plasma concentrations obtained 10 min following intranasal instillation and those immediately after intravenous administration led us to perform a sample size calculation. WinPepi (Abrahmson, version 11.50, 2015) was used to determine the sample size. A minimum of three patients per group was required to achieve a 5% significance level and 80% power.

Data were recorded as medians and interquartile ranges (IQR) owing to asymmetric distribution, except for sex (percentage), which was the only categorical variable. Percentages were compared using Fisher's exact test (2-sided), whereas the median and IQRs were analyzed using the Mann-Whitney U test. Spearman's correlation coefficient was utilized to evaluate the relationship between the actual midazolam doses administered and plasma medication levels. Statistical (p-value) was set at p<0.05. IBM SPSS Statistics ver. 24 (IBM Corp., Armonk, NY, USA) was used for all analyses.

## RESULTS

**Figure 1:**
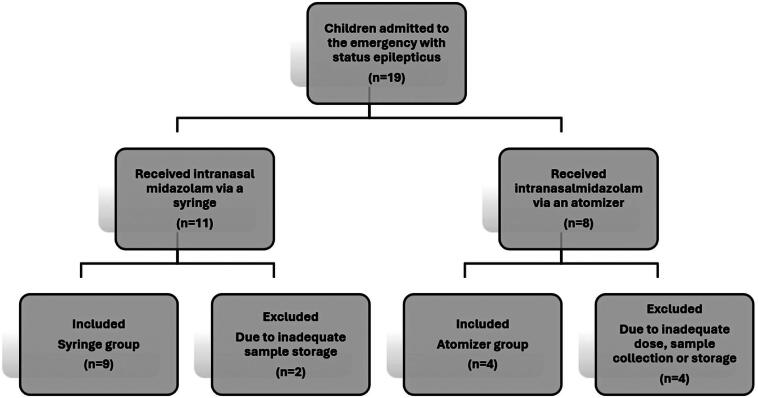
Study indicating the included and excluded patients

Figure 1 shows the study flowchart, detailing the number of included and excluded patients, along with the reasons for exclusion.

Table 1 compares the different clinical features of the patients who received intranasal midazolam via syringe instillation and those who received it using an atomizer. No significant correlation was observed between the actual midazolam doses administered and the plasma medication concentrations (p=0.195; R=-0.384).

**Table 1:** Comparison of clinical and demographic characteristics between patients who received intranasal midazolam via instillation by a syringe and those who received it by atomizer

Characteristics	Syringe (n=9)	Atomizer (n=4)	p value
Sex (males:females)	3:6	3:1	0.266
Age (years)	5.8 (3.9-9.1)	4.6 (2.4-7.2)	0.330
Weight (kg)	17.1 (15.3-24.7)	13.4 (11.0-17.3)	0.148
Current antiseizure medication use, n (%)	6 (66)	2 (50)	0.592
Midazolam dose (mg)	8.5 (7.5-12.2)	7.25 (5.5-8.6)	0.199
Dose according to weight (mg/kg)	0.48 (0.44-0.50)	0.49 (0.47-0.56)	0.414
Time from administration to sample (min)	8.0 (7.75-10.0)	9.5 (6.75-10.0)	0.710
Plasma midazolam concentration (ng/mL)	148.8 (126.3-293.7)	133.3 (76.4-176.3)	0.414

Percentages were compared using Fisher's exact test (two-sided), and medians with interquartile ranges were compared using the Mann-Whitney U test.

Data is expressed as median and interquartile range (IQR), except for the sex and current antiseizure medication use (percentage).

All children treated with midazolam had a prior suspicion or diagnosis of epilepsy. Two-thirds of those in the syringe group and half of those in the atomizer group were already taking antiseizure medications; table 1 depicts this non-significant difference. In the syringe group, three patients had refractory epilepsy due to brain injuries (two of them had congenital malformations) and were receiving carbamazepine, levetiracetam, and clobazam. One child with epilepsy due to the same cause was taking valproate; and the other two patients, whose epilepsy was under investigation, were receiving levetiracetam and carbamazepine, respectively. One child in the atomizer group with brain palsy was receiving carbamazepine, whereas the other child under investigation was receiving valproate. No patient required a second midazolam dose because status epilepticus immediately ceased following midazolam administration in all cases.

## DISCUSSION

This study aimed to address a common issue in pediatric emergencies: whether rapid intranasal midazolam administration via syringe is comparable to delivery via a mucosal atomizer device. Although not a clinical trial, this was an exploratory study addressing a research gap using pharmacokinetic parameters. Our results indicated no significant difference in the peak plasma midazolam concentrations between the two deliveries of intranasal medication.

These findings supports the use of rapid intranasal midazolam delivery via a syringe, an entrenched practice in pediatric emergencies in Brazil and likely in other developing countries. From pharmacoeconomic and social impact perspectives, this approach helps avoid additional costs for healthcare systems that are already stretched to meet increasing demands.

The decision to evaluate the peak plasma concentration approximately 10 min after administration^(13,14)^ was based on existing literature and has clinically relevance. Tonic-clonic status epilepticus is a medical emergency that necessitates swift intervention and control to prevent brain damage.^(2-4)^ The optimal time to decide whether medication is required falls around this 10-min mark.^(4,16)^

Most medications are absorbed from the nasal cavity into the bloodstream within a few minutes, and swallowing may facilitate a secondary absorption phase in the gastrointestinal mucosa,^(11)^ potentially contributing to a sustained anti-seizure effect.

This study had some limitations. The sample size was insufficient for a clinical trial, which prevented us from assessing clinical outcomes. Additionally, the small sample size may account for the lack of correlation between the actual midazolam doses administered and plasma concentrations; however, this was not the main focus of our study. Patient enrollment was particularly challenging because of the strict inclusion criteria, making a large sample size impractical. Furthermore, we lacked data on the final step of midazolam delivery, which could have been obtained through analysis of the cerebrospinal fluid. Finally, the lack of information regarding the duration of status epilepticus before midazolam administration should be recognized as a limitation of this study.

Despite these limitations, this was a crucial exploratory study. To the best of our knowledge, this is the first study to validate such a common practice in Brazilian pediatric emergencies and possibly in other countries worldwide. Further research may contribute to investigating the clinical outcomes of this practice. The ideal design should be a randomized controlled trial, although this is challenging to perform because of ethical and logistical concerns in an emergency setting.
